# A case of Henoch-Schönlein purpura associated with scrub typhus

**DOI:** 10.1186/s12879-020-05001-x

**Published:** 2020-04-17

**Authors:** Jae Hyoung Im, Suk Jin Choi, Moon-Hyun Chung, Seung Yun Lee, Young Kyoung Park, Hea Yoon Kwon, Ji Hyeon Baek, Jin-Soo Lee

**Affiliations:** 1 Division of Infectious Diseases, Department of Internal Medicine, Inha University College of Medicine, 7-206, Shinheung-Dong, Jung-Gu, Incheon, 22332 Republic of Korea; 2 Department of Pathology, Inha University College of Medicine, Incheon, Republic of Korea; 3 Department of Internal Medicine, Seogwipo Medical Center, Jeju, Jeju-do Republic of Korea; 4 Division of Rheumatology,Department of Internal Medicine, Inha University College of Medicine, Incheon, Republic of Korea; 5 Translation Research Center, Inha University College of Medicine, Incheon, Republic of Korea

**Keywords:** Cutaneous, Leukocytoclastic, *Orientia tsutsugamushi*, Henoch-Schönlein, Scrub typhus, Case report

## Abstract

**Background:**

Henoch-Schönlein purpura (HSP) may be caused by several allergens. However, to date, HSP caused by *Orientia tsutsugamushi* has not been reported. Here, we report an unusual rash with features of HSP caused by *Orientia tsutsugamushi*.

**Case presentation:**

A man visited a tertiary hospital with bilateral symmetrical purpura and fever. He presented with an eschar in the left popliteal fossa and proteinuria. He was diagnosed with tsutsugamushi disease by indirect fluorescent antibody and positive polymerase chain reaction tests. Purpura biopsy demonstrated a feature of leukocytoclastic vasculitis and IgA deposition in dermal vessels, indicative of HSP.

**Conclusions:**

When examining patients with unique rashes, such as in this case, we suggest investigating out-door activities and evidence of mite bites. Furthermore, differential diagnosis of tsutsugamushi disease should be considered when necessary.

## Background

Scrub typhus is a febrile disease caused by *Orientia tsutsugamushi (O. tsutsugamushi)*, and is transmitted by infected Leptotrombidium mites [1]. It is characterized by fever, lymphadenopathy, rash, and typical eschar. It causes various complications such as bronchitis, pneumonia, myocarditis, and meningitis [2]. Endothelial activation by *Orientia tsutsugamushi* induces the immune response by promoting the production of interleukins and tumor necrosis factor, which can manifest as various symptoms [3]. Henoch-Schönlein purpura (HSP) is a relatively common vasculitis associated with or induced by infection [4]; however, HSP due to scrub typhus has not been reported. Here, we describe a patient who presented with HSP and an atypical rash, potentially triggered by scrub typhus.

## Case presentation

**Fig. 1:**
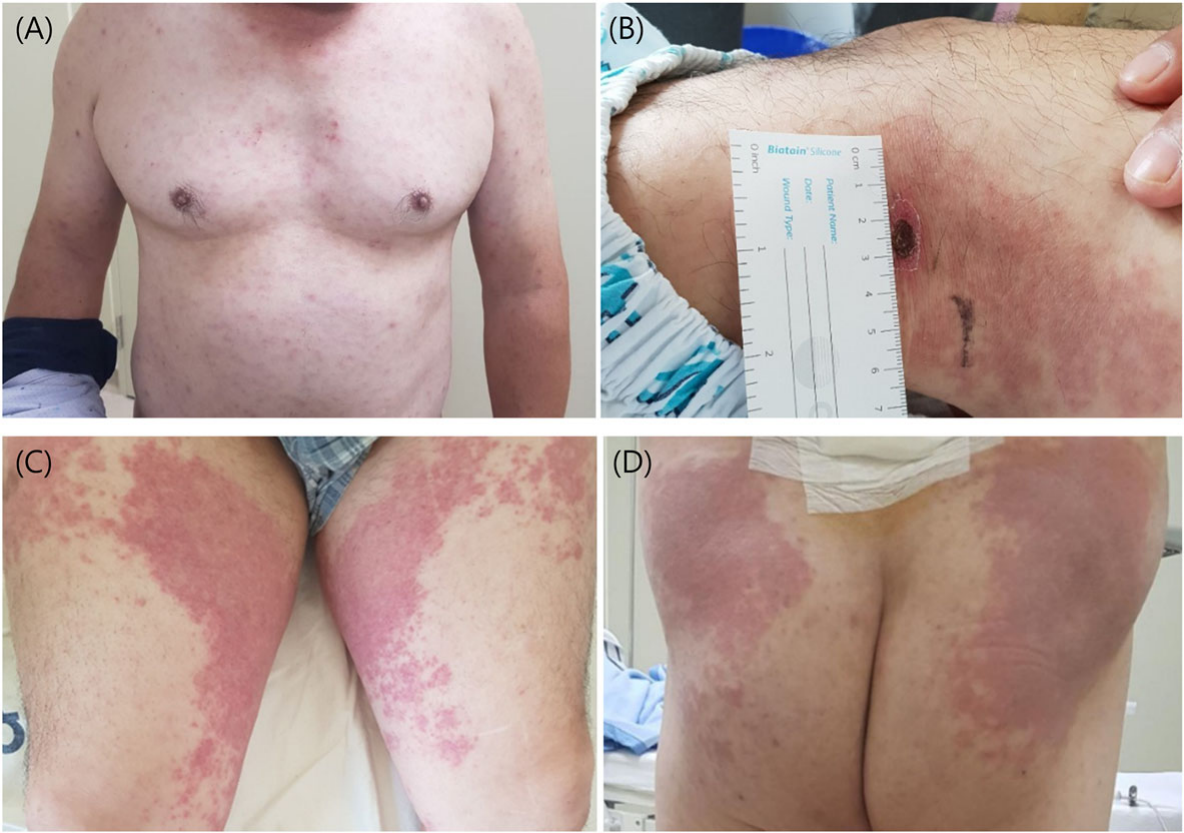
Images show (**a**) maculopapular rash on the trunk, (**b**) eschar on the left popliteal fossa, (**c**) palpable purpura on both lower limbs, and (**d**) palpable purpura on both buttocks

**Fig. 2:**
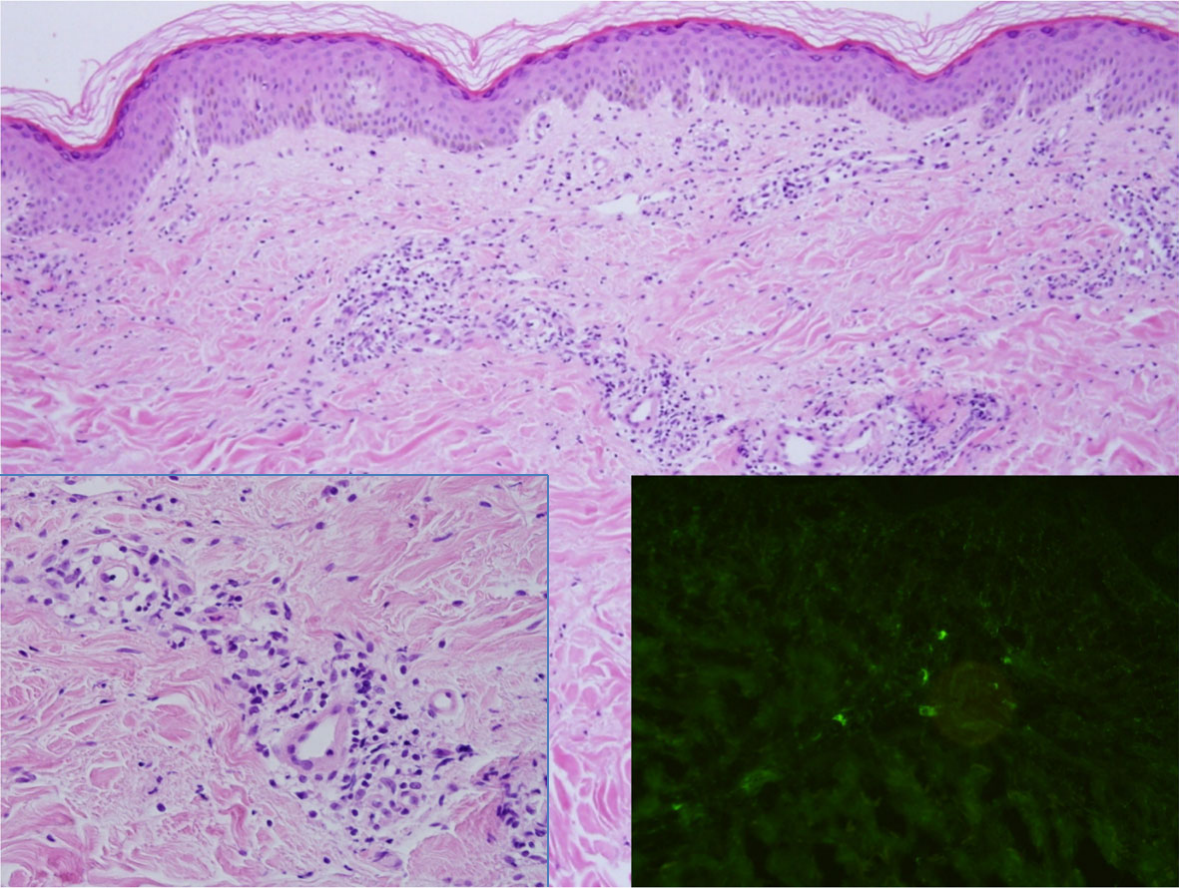
The biopsy of the skin lesion showed perivascular infiltration of inflammatory cells in the upper dermis along with conspicuous karyorrhectic debris (original magnification × 100; *left lower Insert, × 400*) and the presence of IgA deposition on the wall of the blood vessels (direct immunofluorescence test, *right lower insert, × 200*)

A 52-year-old man was admitted to a tertiary hospital (Incheon, Republic of Korea) due to a febrile rash in November. He had diabetes mellitus and hypertension without any complications. At the time of his visit, his blood pressure was 140/90 mmHg, body temperature was 39.9 °C, and heart rate was 107 beats/minute. He did not exhibit any respiratory or gastro-intestinal symptoms or arthralgia. He presented with eschar in the left popliteal fossa. He had a maculopapular rash on his trunk, and palpable purpura was remarkable in the lower extremities (Fig. 1). His leukocyte count was 11,840 cells/μL, hemoglobin was 14.5 g/dL, and platelets were 274,000 cells/μL. The aspartate transaminase/alanine transaminase ratio was slightly elevated to 55/85 IU/L and total bilirubin level (0.8 mg/dL) was normal. The patient had elevated erythrocyte sedimentation rate (22 mm/hour) and C-reactive protein (9.57 mg/dL) values. The urine dipstick test showed proteinuria(++) without pyuria or bacteriuria, and autoantibodies were negative. Although IgM (indirect fluorescent antibody, 1:64), IgG titer (1: 256), and western blot were positive for Lyme disease (Borrelia serology, tested by Korea Center for Diseases Control and prevention [5]), the patient did not present erythema migrans or arthralgia, which are typical findings of Lyme disease. Six weeks later, Borrelia IgM was negative and IgG titer decreased to 1:64, in the Lyme disease test. *O. tsutsugamushi* IgM and IgG titers (indirect fluorescent antibody, Supplementary 1) were 1:1024 and 1:2048, respectively. Further, *O. tsutsugamushi* IgA titer was also high (1:1024). The 56 kDa antigen gene of *O. tsutsugamushi* was positive by polymerase chain reaction on the first day at the hospital (Supplementary 1). Histological examination of the skin biopsy of the rash showed findings of leukocytoclastic vasculitis and deposition of IgA on dermal blood vessel walls (Fig. 2). Rash, histologic findings, and proteinuria satisfied the criteria for diagnosis of Henoch-Schönlein purpura. He had not started any new medications for a month and had no symptoms of any infections other than scrub typhus. Thus, the patient was diagnosed with Henoch-Schönlein purpura associated with scrub typhus. Despite no immune suppressive treatment, the patient showed a dramatic response to doxycycline (100 mg twice/day) alone. His fever resolved within 24 h, and the rash (both maculopapular rash on his trunk and palpable purpura on his lower extremities) began to improve within 48 h. He was discharged after 4 days. Five weeks later, his rash and proteinuria improved, and he is currently living without any sequelae.

## Discussion and conclusions

The case described herein presented without the typical arthritis or abdominal pain of the classic triad of HSP. However, the 2010 European League Against Rheumatism criteria were satisfied [6]*.* There have been several cases of HSP associated with *Rickettsia* spp. [7] and *Bartonella* spp. [8], which are believed to share a common ancestor with *O. tsutsugamushi*. However, scrub typhus-induced HSP has not been reported to date.

Although the pathogenesis of HSP is unclear, it is well known that 30 to 50% of patients are predisposed to upper respiratory tract infection. *Streptococcus, mycoplasma,* hepatitis B virus, herpesvirus, parvovirus, coxsackievirus, adenovirus, measles, and rubella are known to cause HSP [9].*,* In addition to infection, other allergens such as vaccination, drugs, food, and insect bites are also considered as causative agents of HSP [10]. The main pathophysiology of HSP is the presence of abnormal immunoglobulin A (IgA) deposits on the vessel wall [11]. Our patient presented with elevated serum *O. tsutsugamushi* IgA antibodies (1:1024) and there was evidence of IgA deposition based on histopathology evaluation. This suggested that the elevated IgA was due to *O. tsutsugamushi* caused by vasculitis. Aberrant glycosylation of IgA1 is thought to cause HSP [12], and various bacterial and viral pathogens are known to produce sialidase (neuraminidase) [13]. Decreases in sialic acid and Gal may affect IgA1 molecules, leading to the immunological mechanisms of HSP [14]. IgA1 molecules lacking sialic acid or Gal tend to aggregate to form macromolecular complexes, which activate some cytokines and the complement system, and adversely affect the endothelium [14]. In addition, sialic acid-deficient IgA tends to be preferably deposited in the kidneys [11].

In this case, the seropositivity of the Lyme disease test can lead to confusion in the interpretation of the patient’s clinical features. There are three possibilities for seropositivity in Lyme disease: i) co-infection, ii) false positivity in the Lyme disease test, or iii) recrudescence of Lyme disease. Of these, the possibility of co-infection and recrudescence was very low in our case, considering the low incidence of Lyme disease in South Korea [15] and the absence of erythema migrans and arthralgia. The decrease in IgG titer from 1: 256 to 1:64 in 6 weeks also suggests no acute Lyme disease. A previous report showed that extremely high serum IgM levels induced by other pathogens could produce false positive results in the serology testing for Lyme disease (including western blot) [16]. In this case, there was a very high titer of IgM, which could have resulted from the seropositivity for scrub typhus.

This case was accompanied by eschar, which is a typical finding of *O. tsutsugamushi*. Antibiotics were administered promptly, even before the pathogen could be confirmed. However, depending on the patient’s skin color and immunity, no or small-sized eschar may often be encountered in clinical practice [17]. Thus, it is important to identify the potential exposure to vectors and outdoor activities of patients presenting with HSP. Early use of anti-microbial agents such as doxycycline, chloramphenicol, and azithromycin can be imperative in preventing the severity and complications related to scrub typhus.

In conclusion, we report a patient presenting with HSP, potentially triggered by *O. tsutsugamushi*. To facilitate early intervention and prevent complications it is important to consider the possibility of infections such as scrub typhus in the differential diagnosis of unusual rashes.

## Supplementary information

**Additional file 1.**

## Data Availability

The datasets used during the current study are available from the corresponding author upon reasonable request.

## Abbreviations

HSP: Henoch-Schönlein purpura
Ig: Immunoglobulin
