# Supplementary material for: A case of Henoch-Schönlein purpura associated with scrub typhus
Source: BMC Infect Dis. 2020 Apr 17;20:286. doi: 10.1186/s12879-020-05001-x (PMC7165380; doi:10.1186/s12879-020-05001-x)
Supplement: Supplementary file 1 — Additional file 1. [file 12879_2020_5001_MOESM1_ESM.docx]

**Supplementary 1**

<IFA protocol>

Indirect fluorescent antibody test was performed using in-house antigen-coated slide. Briefly, O. tsutsugamushi infected Vero cells were fixed on the glass slide. Serially diluted patient's serum was dropped onto the fixed cells and incubated at 37'C for 30 min. Slide was washed three times with PBS and dried at room temperature. FITC-conjugated secondary antibody for human IgG, IgM, and IgA (Jackson ImmunoReaserch Lab, USA) was dropped onto the slide and incubated at 37'C for 30 min. After washing with PBS three times, mounting oil was dropped onto the dried slide. Slide was covered with a coverglass and observed using a fluorescence microscope (ZEISS Axioscope 2, Germany).

<PCR protocol>

Primer sets used were p34 (forward primer, 5’-TCAAGCTTATTGCTAGTGCAATGTCTGC-3’) and p55 (reverse primer, 5’-AGGGATCCCTGCTGCTGTGCTTGCTGCG-3’). The PCR conditions consisted of an initial denaturation at 94°C for 7min and 30 cycles of 1 min 94°C, 1 min at 57°C, and 1 min at 72°C, with a final extension of 10 min at 70°C.

(Ref: Furuya Y, Yoshida Y, Katayama T, Yamamoto S, Kawamura A, Jr. Serotype-specific amplification of Rickettsia t s utsugamushi DNA by nested polymerase chain reaction. J Clin Microbiol . 1993;31:1637-40.)
